# 3-(2,4-Dichloro­phen­yl)-2-oxo-1-oxaspiro­[4.5]dec-3-en-4-yl acetate

**DOI:** 10.1107/S1600536809053951

**Published:** 2009-12-19

**Authors:** Jin-hao Zhao, Yong Zhou, Jing-Li Cheng, Chuan-Ming Yu, Guo-Nian Zhu

**Affiliations:** a Institute of Pesticide and Environmental Toxicology, Zhejiang University, Hangzhou 310029, People’s Republic of China; b College of Chemical Engineering and Materials Science, Zhejiang University of Technology, Hangzhou 310032, People’s Republic of China

## Abstract

In the title compound, C_17_H_16_Cl_2_O_4_, the cyclo­hexyl ring displays a chair conformation [the four C atoms are planar with a mean deviation of 0.001 (2) Å and the two C atoms at the flap positions deviate by 0.625 (2) and −0.680 (2) Å from the plane]. The furan ring is planar with a mean deviation of 0.004 (2) Å and forms a dihedral angle of 46.73 (2)° with the benzene ring.

## Related literature

For tetronic acid, see: Fischer *et al.* (1993 ▶); Benson *et al.* (2000 ▶). For the chemistry of tetronic acid pesticides, see: BAYER Aktiengesellschaft (1995 ▶). For the synthesis and basic structure of the spiro­diclofen derivative, see: Zhao *et al.* (2009 ▶); Zhou *et al.* (2009 ▶).
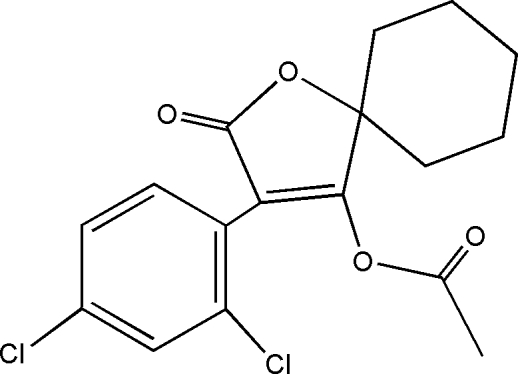


## Experimental

### 

#### Crystal data

C_17_H_16_Cl_2_O_4_
*M* *_r_* = 355.20
Monoclinic, 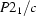

*a* = 14.0705 (5) Å
*b* = 12.9731 (4) Å
*c* = 9.2400 (3) Å
β = 90.8920 (10)°
*V* = 1686.45 (10) Å^3^
*Z* = 4
Mo *K*α radiation
μ = 0.40 mm^−1^
*T* = 296 K
0.47 × 0.45 × 0.29 mm

#### Data collection

Rigaku R-AXIS RAPID diffractometer
Absorption correction: multi-scan (*ABSCOR*; Higashi, 1995 ▶) *T*_min_ = 0.834, *T*_max_ = 0.893
16146 measured reflections
3835 independent reflections
2866 reflections with *I* > 2σ(*I*)
*R*_int_ = 0.025

#### Refinement

*R*[*F* ^2^ > 2σ(*F* ^2^)] = 0.035
*wR*(*F* ^2^) = 0.098
*S* = 1.00
3835 reflections
210 parameters
H-atom parameters constrained
Δρ_max_ = 0.22 e Å^−3^
Δρ_min_ = −0.23 e Å^−3^

### 

Data collection: *PROCESS-AUTO* (Rigaku, 2006 ▶); cell refinement: *PROCESS-AUTO*; data reduction: *CrystalStructure* (Rigaku, 2007 ▶); program(s) used to solve structure: *SHELXS97* (Sheldrick, 2008 ▶); program(s) used to refine structure: *SHELXL97* (Sheldrick, 2008 ▶); molecular graphics: *ORTEP-3 for Windows* (Farrugia, 1997 ▶); software used to prepare material for publication: *WinGX* (Farrugia, 1999 ▶) and *PLATON* (Spek, 2009 ▶).

## Supplementary Material

Crystal structure: contains datablocks I, global. DOI: 10.1107/S1600536809053951/si2227sup1.cif

Structure factors: contains datablocks I. DOI: 10.1107/S1600536809053951/si2227Isup2.hkl

Additional supplementary materials:  crystallographic information; 3D view; checkCIF report

## Figures and Tables

**Fig. 1.**
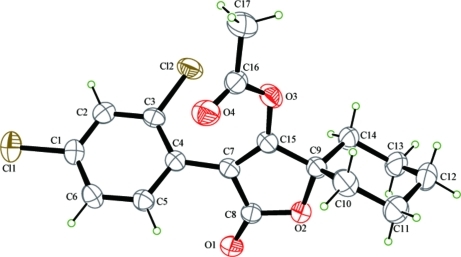
The molecular structure of (I), showing the atom-labelling scheme. Displacement ellipsoids are drawn at the 30% probability level.

## supplementary crystallographic information

### Comment

The chemistry of tetronic acid compounds has being receiving increasing attention in recent years, and references cited therein (Fischer *et al.*,1993; Benson *et al.*, 2000). Bayer company have developed three tetronic acids pesticides-spirodiclofen, spiromesifen and spirotetramat(BAYER Aktiengesellschaft, 1995). The cyclohexyl chair is linked by the spiro carbon atom to the five membered furan ring and the dichlorophenyl group to form the basic structure of the spirodiclofen derivative (Zhao *et al.*, 2009) resulting in the title compound (I), (Fig. 1) by addition of the acetate group. The furan ring is planar with a mean deviation of 0.004 (2) Å. The dihedral angle between benzene and furan rings is 46.73 (2) °. The cyclohexyl ring displays a chair conformation with the deviations of C9 and C12 being 0.625 (2) and -0.680 (2) Å, respectively. Similar distortions were observed in the structure of a spirodiclofen derivative. (Zhou *et al.*, (2009)). As expected, C7=C15, C8=O1 and C16=O4 are typically double bonds with bond distances of 1.336 (2), 1.201 (2) and 1.183 (2) Å, respectively. In the crystal, the molecules are linked through weak intermolecular contacts of C17—H17B···O1, forming chains running along the *c* axis.

### Experimental

4-hydroxyl-3-(2,4-dichlorophenyl)-1-oxaspiro[4,5]dec- 3-en-2-one(10 mmol 3.12 g) was added to acetic anhydride (35 ml) and the mixture was stirred at reflux for 5 h. Then water (70 ml) was added and the solution was extracted with dichloromethane. The organic layer was dried over Na_2_SO_4_. After filtered and concentrated, the organic residue was purified by silica gel column chromatography, eluted with ethyl acetate-petroleum(1:30,*v*/*v*) to give a white solid, which was then recrystallized from 95% ethanol to give colourless blocks.

### Refinement

H atoms were included in calculated positions and refined using a rinding model, with C—H distances constrained to 0.96 Å for methyl H atoms, 0.93Å for aryl H atoms and 0.97 for the cyclopentane,with O—H distances constrained to 0.820 Å, and with *U*_iso_(H) = 1.2Ueq(C,O).

### Crystal data

**Table:**

C_17_H_16_Cl_2_O_4_	*F*(000) = 736
*M**_r_* = 355.20	*D*_x_ = 1.399 Mg m^−^^3^
Monoclinic, *P*2_1_/*c*	Mo *K*α radiation, λ = 0.71073 Å
Hall symbol: -P 2ybc	Cell parameters from 11608 reflections
*a* = 14.0705 (5) Å	θ = 3.1–27.4°
*b* = 12.9731 (4) Å	µ = 0.40 mm^−^^1^
*c* = 9.2400 (3) Å	*T* = 296 K
β = 90.892 (1)°	Chunk, colorless
*V* = 1686.45 (10) Å^3^	0.47 × 0.45 × 0.29 mm
*Z* = 4	

### Data collection

**Table:**

Rigaku R-AXIS RAPID diffractometer	3835 independent reflections
Radiation source: rotating anode	2866 reflections with *I* > 2σ(*I*)
graphite	*R*_int_ = 0.025
Detector resolution: 10.00 pixels mm^-1^	θ_max_ = 27.4°, θ_min_ = 3.1°
ω scans	*h* = −17→18
Absorption correction: multi-scan (*ABSCOR*; Higashi, 1995)	*k* = −16→16
*T*_min_ = 0.834, *T*_max_ = 0.893	*l* = −11→11
16146 measured reflections	

### Refinement

**Table:**

Refinement on *F*^2^	Secondary atom site location: difference Fourier map
Least-squares matrix: full	Hydrogen site location: inferred from neighbouring sites
*R*[*F*^2^ > 2σ(*F*^2^)] = 0.035	H-atom parameters constrained
*wR*(*F*^2^) = 0.098	*w* = 1/[σ^2^(*F*_o_^2^) + (0.040*P*)^2^ + 0.650*P*] where *P* = (*F*_o_^2^ + 2*F*_c_^2^)/3
*S* = 1.00	(Δ/σ)_max_ = 0.001
3835 reflections	Δρ_max_ = 0.22 e Å^−^^3^
210 parameters	Δρ_min_ = −0.23 e Å^−^^3^
0 restraints	Extinction correction: *SHELXL97* (Sheldrick, 2008), Fc^*^=kFc[1+0.001xFc^2^λ^3^/sin(2θ)]^-1/4^
Primary atom site location: structure-invariant direct methods	Extinction coefficient: 0.0064 (10)

### Special details

**Table:**

Geometry. All e.s.d.'s (except the e.s.d. in the dihedral angle between two l.s. planes) are estimated using the full covariance matrix. The cell e.s.d.'s are taken into account individually in the estimation of e.s.d.'s in distances, angles and torsion angles; correlations between e.s.d.'s in cell parameters are only used when they are defined by crystal symmetry. An approximate (isotropic) treatment of cell e.s.d.'s is used for estimating e.s.d.'s involving l.s. planes.
Refinement. Refinement of *F*^2^ against ALL reflections. The weighted *R*-factor *wR* and goodness of fit *S* are based on *F*^2^, conventional *R*-factors *R* are based on *F*, with *F* set to zero for negative *F*^2^. The threshold expression of *F*^2^ > σ(*F*^2^) is used only for calculating *R*-factors(gt) *etc*. and is not relevant to the choice of reflections for refinement. *R*-factors based on *F*^2^ are statistically about twice as large as those based on *F*, and *R*- factors based on ALL data will be even larger.

### Fractional atomic coordinates and isotropic or equivalent isotropic displacement parameters (Å^2^)

**Table:**

	*x*	*y*	*z*	*U*_iso_*/*U*_eq_	
Cl2	0.54892 (4)	0.47091 (4)	0.18788 (5)	0.06065 (17)	
Cl1	0.38311 (4)	0.75742 (4)	0.51693 (6)	0.06588 (18)	
O2	0.84926 (8)	0.39184 (10)	0.46610 (12)	0.0476 (3)	
O1	0.75521 (9)	0.45025 (11)	0.64035 (13)	0.0536 (3)	
C4	0.64126 (12)	0.56385 (12)	0.41374 (17)	0.0392 (3)	
O3	0.76541 (10)	0.49671 (10)	0.13019 (13)	0.0549 (3)	
C16	0.74521 (13)	0.59698 (16)	0.08843 (19)	0.0490 (4)	
C7	0.72584 (12)	0.49944 (13)	0.39001 (17)	0.0401 (4)	
C6	0.56414 (13)	0.69385 (14)	0.5600 (2)	0.0500 (4)	
H6	0.5665	0.7404	0.6365	0.060*	
C15	0.77593 (13)	0.47332 (13)	0.27392 (18)	0.0437 (4)	
C3	0.55724 (12)	0.55682 (13)	0.33197 (17)	0.0417 (4)	
C1	0.48284 (12)	0.68374 (13)	0.4763 (2)	0.0462 (4)	
C9	0.85752 (12)	0.40299 (14)	0.30992 (18)	0.0445 (4)	
C2	0.47789 (12)	0.61566 (14)	0.36203 (18)	0.0460 (4)	
H2	0.4225	0.6094	0.3064	0.055*	
O4	0.74584 (11)	0.66581 (11)	0.17247 (15)	0.0626 (4)	
C5	0.64214 (13)	0.63370 (14)	0.52862 (19)	0.0467 (4)	
H5	0.6969	0.6399	0.5857	0.056*	
C8	0.77430 (12)	0.44761 (13)	0.51412 (18)	0.0424 (4)	
C14	0.84764 (13)	0.29681 (15)	0.2402 (2)	0.0522 (4)	
H14A	0.7892	0.2649	0.2718	0.063*	
H14B	0.8438	0.3044	0.1358	0.063*	
C13	0.93139 (16)	0.22725 (18)	0.2799 (3)	0.0715 (6)	
H13A	0.9306	0.2126	0.3829	0.086*	
H13B	0.9252	0.1624	0.2284	0.086*	
C10	0.95350 (14)	0.45228 (17)	0.2795 (2)	0.0620 (5)	
H10A	0.9596	0.5155	0.3348	0.074*	
H10B	0.9564	0.4699	0.1776	0.074*	
C11	1.03604 (15)	0.3808 (2)	0.3184 (3)	0.0772 (7)	
H11A	1.0954	0.4127	0.2907	0.093*	
H11B	1.0382	0.3700	0.4223	0.093*	
C17	0.72453 (19)	0.6010 (2)	−0.0704 (2)	0.0764 (7)	
H17A	0.6580	0.5890	−0.0877	0.092*	
H17B	0.7608	0.5488	−0.1184	0.092*	
H17C	0.7415	0.6676	−0.1071	0.092*	
C12	1.02541 (16)	0.2775 (2)	0.2422 (3)	0.0867 (8)	
H12A	1.0774	0.2326	0.2710	0.104*	
H12B	1.0282	0.2876	0.1383	0.104*	

### Atomic displacement parameters (Å^2^)

**Table:**

	*U*^11^	*U*^22^	*U*^33^	*U*^12^	*U*^13^	*U*^23^
Cl2	0.0609 (3)	0.0749 (3)	0.0461 (3)	−0.0062 (2)	−0.0021 (2)	−0.0182 (2)
Cl1	0.0520 (3)	0.0588 (3)	0.0873 (4)	0.0111 (2)	0.0151 (2)	−0.0012 (3)
O2	0.0461 (6)	0.0517 (7)	0.0450 (6)	0.0078 (6)	−0.0016 (5)	−0.0027 (5)
O1	0.0572 (8)	0.0654 (8)	0.0381 (6)	0.0063 (6)	−0.0007 (5)	−0.0010 (6)
C4	0.0433 (8)	0.0371 (8)	0.0374 (8)	−0.0017 (7)	0.0032 (7)	0.0010 (6)
O3	0.0725 (9)	0.0540 (7)	0.0385 (6)	0.0110 (7)	0.0104 (6)	−0.0009 (5)
C16	0.0452 (9)	0.0568 (11)	0.0450 (9)	0.0032 (8)	0.0047 (7)	0.0058 (9)
C7	0.0452 (9)	0.0375 (8)	0.0374 (8)	−0.0028 (7)	0.0015 (7)	−0.0027 (7)
C6	0.0523 (10)	0.0443 (9)	0.0535 (10)	−0.0017 (8)	0.0075 (8)	−0.0110 (8)
C15	0.0502 (9)	0.0409 (9)	0.0401 (8)	0.0013 (7)	0.0035 (7)	−0.0017 (7)
C3	0.0483 (9)	0.0428 (9)	0.0341 (8)	−0.0050 (7)	0.0027 (7)	0.0017 (7)
C1	0.0441 (9)	0.0409 (9)	0.0540 (10)	0.0017 (7)	0.0110 (8)	0.0058 (8)
C9	0.0434 (9)	0.0462 (9)	0.0439 (9)	0.0007 (7)	0.0021 (7)	−0.0073 (7)
C2	0.0423 (9)	0.0514 (10)	0.0444 (9)	−0.0025 (8)	0.0017 (7)	0.0078 (8)
O4	0.0823 (10)	0.0506 (8)	0.0545 (8)	0.0025 (7)	−0.0065 (7)	0.0040 (7)
C5	0.0449 (9)	0.0472 (10)	0.0478 (9)	−0.0006 (8)	−0.0014 (7)	−0.0085 (8)
C8	0.0417 (8)	0.0409 (9)	0.0445 (9)	−0.0021 (7)	−0.0019 (7)	−0.0037 (7)
C14	0.0444 (9)	0.0498 (10)	0.0621 (11)	0.0047 (8)	−0.0058 (8)	−0.0140 (9)
C13	0.0658 (13)	0.0610 (13)	0.0869 (16)	0.0216 (11)	−0.0206 (12)	−0.0258 (12)
C10	0.0538 (11)	0.0662 (13)	0.0662 (12)	−0.0137 (10)	0.0109 (10)	−0.0151 (10)
C11	0.0405 (10)	0.1057 (19)	0.0856 (16)	−0.0067 (11)	0.0009 (10)	−0.0284 (14)
C17	0.0956 (17)	0.0911 (17)	0.0426 (10)	0.0107 (14)	0.0081 (11)	0.0080 (11)
C12	0.0493 (12)	0.112 (2)	0.0985 (18)	0.0272 (13)	−0.0115 (12)	−0.0418 (16)

### Geometric parameters (Å, °)

**Table:**

Cl2—C3	1.7388 (17)	C9—C14	1.526 (2)
Cl1—C1	1.7437 (17)	C2—H2	0.9300
O2—C8	1.359 (2)	C5—H5	0.9300
O2—C9	1.457 (2)	C14—C13	1.525 (3)
O1—C8	1.201 (2)	C14—H14A	0.9700
C4—C5	1.395 (2)	C14—H14B	0.9700
C4—C3	1.396 (2)	C13—C12	1.520 (4)
C4—C7	1.473 (2)	C13—H13A	0.9700
O3—C15	1.368 (2)	C13—H13B	0.9700
O3—C16	1.385 (2)	C10—C11	1.525 (3)
C16—O4	1.183 (2)	C10—H10A	0.9700
C16—C17	1.492 (3)	C10—H10B	0.9700
C7—C15	1.336 (2)	C11—C12	1.520 (3)
C7—C8	1.486 (2)	C11—H11A	0.9700
C6—C1	1.377 (3)	C11—H11B	0.9700
C6—C5	1.381 (2)	C17—H17A	0.9600
C6—H6	0.9300	C17—H17B	0.9600
C15—C9	1.500 (2)	C17—H17C	0.9600
C3—C2	1.384 (2)	C12—H12A	0.9700
C1—C2	1.377 (3)	C12—H12B	0.9700
C9—C10	1.524 (3)		
			
C8—O2—C9	110.17 (12)	O2—C8—C7	109.76 (14)
C5—C4—C3	116.84 (15)	C13—C14—C9	111.57 (15)
C5—C4—C7	118.94 (14)	C13—C14—H14A	109.3
C3—C4—C7	124.16 (15)	C9—C14—H14A	109.3
C15—O3—C16	119.83 (14)	C13—C14—H14B	109.3
O4—C16—O3	121.77 (16)	C9—C14—H14B	109.3
O4—C16—C17	128.21 (19)	H14A—C14—H14B	108.0
O3—C16—C17	110.02 (18)	C12—C13—C14	111.3 (2)
C15—C7—C4	134.48 (15)	C12—C13—H13A	109.4
C15—C7—C8	105.27 (15)	C14—C13—H13A	109.4
C4—C7—C8	120.25 (14)	C12—C13—H13B	109.4
C1—C6—C5	118.93 (16)	C14—C13—H13B	109.4
C1—C6—H6	120.5	H13A—C13—H13B	108.0
C5—C6—H6	120.5	C9—C10—C11	112.02 (18)
C7—C15—O3	132.27 (16)	C9—C10—H10A	109.2
C7—C15—C9	112.81 (15)	C11—C10—H10A	109.2
O3—C15—C9	114.90 (14)	C9—C10—H10B	109.2
C2—C3—C4	122.29 (15)	C11—C10—H10B	109.2
C2—C3—Cl2	117.53 (13)	H10A—C10—H10B	107.9
C4—C3—Cl2	120.18 (13)	C12—C11—C10	110.95 (17)
C6—C1—C2	121.56 (16)	C12—C11—H11A	109.4
C6—C1—Cl1	119.41 (14)	C10—C11—H11A	109.4
C2—C1—Cl1	119.02 (14)	C12—C11—H11B	109.4
O2—C9—C15	101.97 (13)	C10—C11—H11B	109.4
O2—C9—C10	108.00 (14)	H11A—C11—H11B	108.0
C15—C9—C10	112.41 (16)	C16—C17—H17A	109.5
O2—C9—C14	108.69 (15)	C16—C17—H17B	109.5
C15—C9—C14	113.02 (14)	H17A—C17—H17B	109.5
C10—C9—C14	112.08 (15)	C16—C17—H17C	109.5
C1—C2—C3	118.43 (16)	H17A—C17—H17C	109.5
C1—C2—H2	120.8	H17B—C17—H17C	109.5
C3—C2—H2	120.8	C11—C12—C13	110.58 (18)
C6—C5—C4	121.95 (16)	C11—C12—H12A	109.5
C6—C5—H5	119.0	C13—C12—H12A	109.5
C4—C5—H5	119.0	C11—C12—H12B	109.5
O1—C8—O2	121.23 (15)	C13—C12—H12B	109.5
O1—C8—C7	129.01 (16)	H12A—C12—H12B	108.1
			
C15—O3—C16—O4	7.6 (3)	C7—C15—C9—C14	115.89 (18)
C15—O3—C16—C17	−172.61 (17)	O3—C15—C9—C14	−62.8 (2)
C5—C4—C7—C15	−134.5 (2)	C6—C1—C2—C3	−0.2 (3)
C3—C4—C7—C15	48.4 (3)	Cl1—C1—C2—C3	−179.30 (13)
C5—C4—C7—C8	44.9 (2)	C4—C3—C2—C1	0.4 (3)
C3—C4—C7—C8	−132.16 (17)	Cl2—C3—C2—C1	179.61 (13)
C4—C7—C15—O3	−1.1 (3)	C1—C6—C5—C4	0.7 (3)
C8—C7—C15—O3	179.42 (18)	C3—C4—C5—C6	−0.5 (3)
C4—C7—C15—C9	−179.47 (17)	C7—C4—C5—C6	−177.72 (16)
C8—C7—C15—C9	1.07 (19)	C9—O2—C8—O1	−179.13 (16)
C16—O3—C15—C7	44.5 (3)	C9—O2—C8—C7	0.84 (18)
C16—O3—C15—C9	−137.13 (16)	C15—C7—C8—O1	178.78 (18)
C5—C4—C3—C2	−0.1 (2)	C4—C7—C8—O1	−0.8 (3)
C7—C4—C3—C2	177.04 (15)	C15—C7—C8—O2	−1.19 (19)
C5—C4—C3—Cl2	−179.29 (13)	C4—C7—C8—O2	179.26 (14)
C7—C4—C3—Cl2	−2.2 (2)	O2—C9—C14—C13	−67.2 (2)
C5—C6—C1—C2	−0.3 (3)	C15—C9—C14—C13	−179.60 (18)
C5—C6—C1—Cl1	178.78 (14)	C10—C9—C14—C13	52.1 (2)
C8—O2—C9—C15	−0.20 (17)	C9—C14—C13—C12	−55.0 (2)
C8—O2—C9—C10	118.41 (16)	O2—C9—C10—C11	67.5 (2)
C8—O2—C9—C14	−119.78 (15)	C15—C9—C10—C11	179.18 (16)
C7—C15—C9—O2	−0.60 (19)	C14—C9—C10—C11	−52.2 (2)
O3—C15—C9—O2	−179.25 (14)	C9—C10—C11—C12	54.8 (3)
C7—C15—C9—C10	−116.02 (17)	C10—C11—C12—C13	−57.3 (3)
O3—C15—C9—C10	65.3 (2)	C14—C13—C12—C11	57.6 (3)
